# Hypomyelination With Congenital Cataract: A Rare Genetic Leukodystrophy

**DOI:** 10.7759/cureus.92632

**Published:** 2025-09-18

**Authors:** Venkat Meghana Bhimanadham, Gayathri J Panicker

**Affiliations:** 1 Department of Ophthalmology, Sri Ramachandra Institute of Higher Education and Research, Chennai, IND

**Keywords:** autosomal recessive, congenital cataract, fam126a mutation, hypomyelination, india, leukodystrophy, mri brain

## Abstract

Hypomyelination and congenital cataract (HCC) is a rare autosomal recessive disorder characterized by a triad of bilateral cataracts, neurological impairment, and diffuse cerebral hypomyelination. We report a case of a child, born of a consanguineous marriage, who presented with tremors and delayed motor abilities. Clinical examination revealed bilateral lamellar cataracts and microcephaly. MRI brain demonstrated diffuse white matter hypomyelination. The patient underwent cataract surgery with intraocular lens implantation and was managed with supportive rehabilitation. This case highlights the importance of early recognition of ophthalmic manifestations of systemic neurogenetic disorders, the diagnostic role of neuroimaging and genetic testing, and the necessity of multidisciplinary management.

## Introduction

Leukodystrophies are a heterogeneous group of inherited disorders that primarily affect the central nervous system (CNS), characterized by impaired myelin formation, abnormal myelin maintenance, or accelerated demyelination. A rare subset of these disorders is hypomyelinating disorders, where myelin deposition is insufficient rather than degraded.

Hypomyelination with congenital cataract (HCC) is a distinctive leukodystrophy first described in 2006, with fewer than 100 cases reported globally to date [1,2]. Although cases have been described in Europe, North America, and the Middle East, only a very limited number have been reported from India [3]. This underscores the need for increased awareness among ophthalmologists and pediatricians in regions where consanguinity is prevalent. Importantly, early recognition of cataracts is essential to prevent amblyopia, and clinicians should consider ophthalmic screening in children presenting with developmental delay or neurological regression. Multidisciplinary management remains the cornerstone of optimizing outcomes in these complex patients.

The condition has an autosomal recessive pattern of inheritance with mutations in the FAM126A gene located on chromosome 7p15. This gene encodes hyccin, a protein essential for the function of oligodendrocytes and Schwann cells. Deficiency of this enzyme results in impaired myelination in both the CNS and the peripheral nervous system (PNS) [4,5]. Clinically, HCC is characterized by a triad of bilateral congenital cataract, neurological dysfunction, and diffuse white matter hypomyelination on MRI [6,7].

## Case presentation

**Figure 1:**
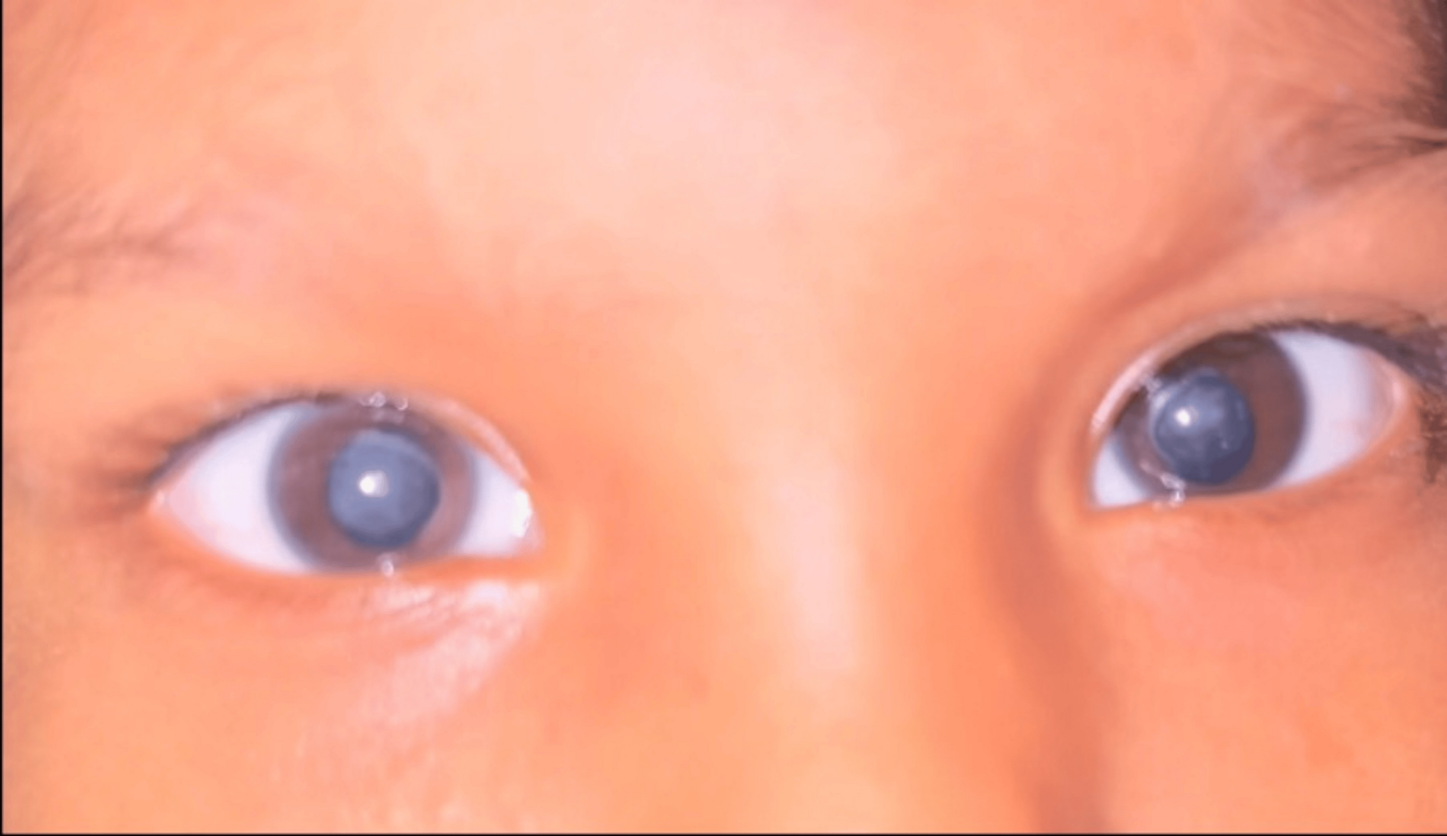
Bilateral lamellar cataract

A previously healthy two-and-a-half-year-old girl, born of a consanguineous marriage, presented to the outpatient department with a 15-day history of tremors while holding objects and an inability to stand or walk. She was admitted to the pediatric intensive care unit in view of worsening tremors and functional regression, which required close monitoring and supportive stabilization. Examination showed microcephaly with a head circumference of 43.5 cm (<-3 Z score). Neurological assessment revealed titubations, mild hypotonia in the lower limbs with a muscle power of 4/5, bilateral brisk knee jerks, and extensor plantar responses. Detailed ophthalmic evaluation was performed, and findings were consistent with bilateral lamellar cataract (right eye > left eye); a variable intermittent squint was observed (Figure 1). Visual acuity in both eyes was 4 cpcm with Lea paddles, and fundus examination showed no obvious retinal pathology. Ocular biometry was performed prior to cataract surgery. Axial length was measured using contact A-scan biometry, and intraocular lens (IOL) power calculation was performed with the SRK/T formula.

**Figure 2:**
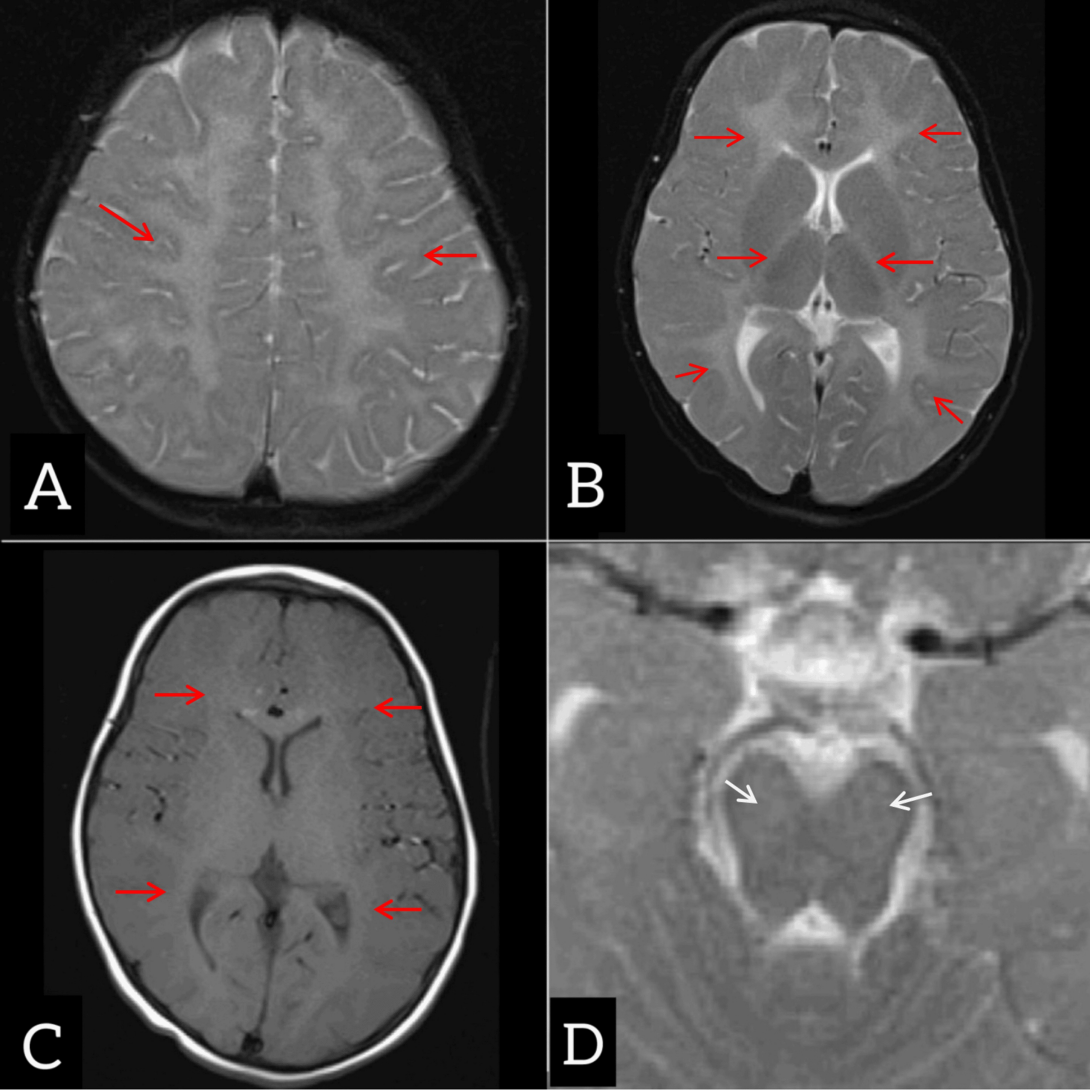
Axial sections of T2-weighted (A and B) and T1-weighted (non-contrast) (C) images showing diffuse hypomyelination of the white matter of bilateral cerebral hemispheres, including the subcortical and deep white matter and bilateral internal capsule (red arrows). Axial sections of T2 WI (D) at the level of the midbrain show patchy, subtle hypomyelination involving the midbrain (white arrows)

MRI of the brain demonstrated diffuse hypomyelination involving subcortical, deep cerebral white matter and internal capsules with mild involvement of the midbrain (Figure 2). MRI spine screening revealed no significant abnormality. Initial laboratory investigations revealed microcytic hypochromic anemia. Nerve conduction studies (NCS) were obtained to rule out peripheral neuropathy, given that HCC may involve both CNS and PNS pathways, despite the presence of upper motor neuron signs. Electroencephalography was performed to exclude subclinical epileptiform activity, which can be seen in leukodystrophies even in the absence of clinical seizures. Genetic testing was done in the form of whole exome sequencing and showed a bi-allelic pathogenic FAM126A variant, thereby confirming the diagnosis of HCC.

The patient underwent bilateral cataract extraction by lens aspiration with posterior capsulorhexis and anterior vitrectomy, followed by IOL implantation under general anesthesia. Rehabilitative therapy was initiated in the form of physiotherapy and occupational therapy for motor development. Microcytic hypochromic anemia was identified and managed with nutritional counseling and oral iron supplementation.

She was followed up three months post-surgery, and improvement in visual acuity was observed. Neurological status was stable with mild tremors and hypotonia. The child continued to receive supportive therapies.

## Discussion

HCC is an extremely rare leukodystrophy, notable for its combination of ocular and neurological features. Recognition of cataracts at an early stage is critical, since ophthalmologists may be the first clinicians to encounter affected children. The classical hallmark triad includes ophthalmological manifestations (bilateral congenital or early-onset cataract, nystagmus, and visual impairment), neurological features (hypotonia, tremors, titubation, spasticity, pyramidal signs, delayed motor development, and eventual loss of ambulation), and MRI findings (suggestive of hypomyelination of supratentorial white matter). The FAM126A gene encodes hyccin, which is involved in phosphatidylinositol 4-kinase trafficking and myelination. Loss-of-function mutations in this gene result in defective oligodendrocyte and Schwann cell activity, manifesting as hypomyelination in the CNS and variable peripheral neuropathy [4,5]. HCC exhibits broad phenotypic heterogeneity. Reports from India are sparse. Gowda et al. described a novel pathogenic variant in FAM126A in an Indian cohort, thereby expanding the phenotypic spectrum [3]. Our report further supports the occurrence of HCC in this population, where consanguinity remains a significant risk factor. Classical cases develop normally in infancy, then progressively decline, while early-onset forms present within the first year with severe disability. Late-onset forms manifest after two years with slower progression [6].

Our patient’s clinical presentation, characterized by visual impairment, neurological manifestations, and MRI findings, was highly consistent with the classic triad of late-onset HCC phenotype. In the differential diagnosis, other neuro-ophthalmologic conditions, such as Pelizaeus-Merzbacher disease and 4H syndrome, as well as metabolic disorders like galactosemia, were considered. Careful clinical correlation, neuroimaging, and genetic testing are necessary for distinction.

In the absence of an established disease-modifying therapy for HCC, management remains largely multidisciplinary and supportive. Early cataract surgery with IOL implantation is essential for visual rehabilitation and preventing amblyopia. Neuro-rehabilitation, comprising physiotherapy and occupational therapy, improves motor outcomes and helps maintain functional independence. Genetic counseling is crucial for affected families, especially in consanguineous marriages, to enlighten them on recurrence risk and potential for prenatal diagnosis. This reinforces the importance of routine ophthalmic screening in children with developmental delay or neurological regression, as early detection of cataracts is one of the few treatable aspects of this otherwise progressive leukodystrophy.

This case contributes to the existing literature by documenting bilateral lamellar cataract with early-onset motor dysfunction, microcephaly, and a genetically confirmed FAM126A pathogenic variant, reinforcing the phenotypic spectrum.

Biancheri et al. described the characteristic triad of bilateral cataract, neurological impairment, and hypomyelination, expanding the clinical phenotype [8]. Troncoso et al. reported novel FAM126A variants and emphasized genetic heterogeneity [9]. Karalok et al. documented three affected siblings, underscoring the role of consanguinity [10]. Additional reports continue to expand the spectrum, with variable severity and progression (Table 1).

**Table 1:** Overview of literature on HCC HCC: hypomyelination and congenital cataract, MRI: magnetic resonance imaging

Author	No. of cases	Age at presentation	Key clinical features	MRI findings	Genetic mutation	Outcome
Zara et al. [1]	4	Infancy	Bilateral cataracts, progressive motor impairment	Diffuse hypomyelination	FAM126A homozygous deletions	Progressive decline
Biancheri et al. [8]	3	1–4 yrs	Cataracts, pyramidal signs, delayed milestones	White matter hypomyelination	FAM126A mutations	Slowly progressive
Gazzerro et al. [5]	10	0.5–6yrs	Cataracts, microcephaly, tremors, peripheral neuropathy	Hypomyelination, internal capsule involvement	FAM126A truncating variants	Variable severity
Karalok et al. [10]	3 siblings	1–3 yrs	Cataracts, delayed motor skills, hypotonia	Diffuse hypomyelination	FAM126A variant	All non-ambulatory
Troncoso et al. [9]	1	2 yrs	Bilateral cataracts, tremors	Hypomyelination including brainstem	Novel FAM126A splice site mutation	Severe neurological impairment

## Conclusions

HCC is a rare but distinct leukodystrophy that should be suspected in children with congenital or early-onset cataracts accompanied by neurological manifestations. Early clinical recognition by ophthalmologists, followed by neuroimaging and confirmatory genetic testing, allows timely intervention and counseling. While treatment remains supportive, visual outcomes can be optimized by prompt cataract surgery and aggressive amblyopia prevention. Rehabilitation, including physiotherapy and occupational therapy, may help preserve motor function. A multidisciplinary approach, uniting ophthalmologists, neurologists, radiologists, rehabilitation specialists, and genetic counselors, is essential for holistic care. In the Indian subcontinent, where consanguinity is common, increased vigilance and family counseling are crucial to early detection and prevention of recurrence.
